# A comprehensive qualitative framework for health-related quality of life in Duchenne muscular dystrophy

**DOI:** 10.1007/s11136-022-03240-w

**Published:** 2022-09-01

**Authors:** Philip A. Powell, Jill Carlton

**Affiliations:** School of Health and Related Research (ScHARR), University of Sheffield, Regent Court, 30 Regent Street, Sheffield, S1 4DA UK

**Keywords:** Duchenne muscular dystrophy, Health-related quality of life, Patient-reported outcomes, Qualitative research, Rare diseases

## Abstract

**Purpose:**

Duchenne muscular dystrophy (DMD) is a rare x-linked neuromuscular condition predominantly affecting boys and men. There is a paucity of research qualitatively detailing the lived experience of health-related quality of life (HRQoL) for people with DMD. The aim of this study was to identify a comprehensive framework for better understanding HRQoL in DMD.

**Methods:**

Eighteen boys and men (aged 7 to ≥ 40 years) with DMD were recruited from charity Duchenne UK, a DMD support group, and 5 UK National Health Service Trusts. Semi-structured interviews were conducted using a topic guide informed by a review into HRQoL in DMD. Generic, preference-based, patient-reported outcome measures (PROs) were used as prompts. Interviews were audio recorded, transcribed verbatim and analysed using framework analysis.

**Results:**

Thirty-seven themes were coded, within seven categories. Six categories were conceptualised as components of HRQoL (autonomy, daily activities, feelings and emotions, identity, physical aspects, social relationships) and one considered an input (healthcare, support, and environment). Three additional themes were used to code feedback on the generic PROs (CHU-9D, EQ-5D, HUI). Social relationships received most coverage in the data and was noted as an omission from the PROs.

**Conclusion:**

A 30-item framework for HRQoL in DMD has been developed, which was used as input into a new condition-specific HRQoL PRO and preference-based measure: the DMD-QoL. The data has value in its own right in highlighting the lived experience of HRQoL for people with DMD and as a barometer for assessing the content of HRQoL PROs for use in DMD.

**Supplementary Information:**

The online version contains supplementary material available at 10.1007/s11136-022-03240-w.

## Plain English Summary

Duchenne muscular dystrophy (DMD) is a rare genetic neuromuscular disorder predominantly affecting boys and men. While studies have suggested health-related quality of life is impaired in people living with DMD, this evidence has been based on responses to questionnaires. There is much less evidence on the lived experience of DMD that is acquired by sitting down and talking to people. The aim of this study was to interview boys and men with DMD to build up a richer and more complete picture of their health-related quality of life, in order to develop a framework that would help further understanding, drive research, and provide a resource for scaffolding future interventions. We also used the findings from this study to develop a new questionnaire for assessing health-related quality of life in DMD (the “DMD-QoL”), based on participants’ own experiences. Thirty different themes important to people living with DMD were identified in the interviews, organised within six categories (autonomy, daily activities, feelings and emotions, identity, physical aspects, social relationships). Of all the domains, social relationships were talked about the most by people in this study and would represent a useful target for future initiatives. This domain may be missing in existing specialised questionnaires that are used to assess health-related quality of life for the cost-effectiveness evaluation of health interventions.

## Introduction

Duchenne muscular dystrophy (DMD) is a rare x-linked neuromuscular condition affecting approximately 19.8 per 100,000 males from birth [1]. DMD is characterised by an absence of dystrophin, a protein vital for muscle cell cohesion. Physical and functional impairment is evident from an early age. In people with the condition, there is progressive muscle weakness, which first affects the proximal muscles, with loss of ambulation and ability to weight bear, followed by loss of upper body function. DMD causes eventual cardiovascular and respiratory complications and results in a reduced life expectancy of a median of 32 years [2]. Presently, there is no cure for DMD, so clinical management involves treating symptoms, attempting to slow progression, and maximising patients’ health-related quality of life (HRQoL). HRQoL has been defined as “a term referring to the health aspects of quality of life, generally considered to reflect the impact of disease and treatment on disability and daily functioning; it has also been considered to reflect the impact of perceived health on an individual’s ability to live a fulfilling life.” [3].

While a number of patient-reported outcome (PRO) tools have been used in DMD [4], and studies suggest HRQoL is impaired relative to general public norms [5–7], there is little published qualitative work describing the richness of HRQoL in people living with DMD. Such evidence is needed to reflect more fully the lived experience of people with DMD and the multitude of ways the condition affects their HRQoL. It provides an accessible resource for stakeholders that details, in participants’ own words, the impact of DMD on their HRQoL, typically including broader issues than that covered quantitatively. Further, a comprehensive qualitative framework of HRQoL in DMD can be used as a benchmark for assessing the content validity of PROs designed to measure the construct. This is important as a previous review has shown that the content validity of the majority of PROs used in DMD has neither been adequately assessed nor evidenced [4].

Qualitative studies that have been published with people living with DMD include: investigations of decision-making and preferences regarding clinical research and treatments [8–12]; experiences of living into adulthood [13–15]; impact on siblings [16]; end of life planning [17]; and specific targeted qualitative investigations into domains that may be considered aspects of, or related to, HRQoL, such as spirituality [18], identity [19], and independence [20, 21]. Further, there are a range of qualitative studies exploring the impact of DMD on caregiver HRQoL outcomes [22]. However, to the authors’ knowledge, no work has been published that characterises comprehensively HRQoL in DMD from the perspective of boys and men with the condition.

In the present study, semi-structured interviews were conducted with boys and men with DMD with the objective of identifying a comprehensive framework for understanding HRQoL in Duchenne. This work was used to support the development of a novel condition-specific, preference-based measure of HRQoL in DMD: the DMD-QoL [23–25], but has significant value in its own right as a rich insight into the lived experiences of people with DMD.

## Methods

### Recruitment and participants

Eighteen boys and men (aged 7 to ≥ 40 years) with a primary diagnosis of DMD were recruited for this study from charity Duchenne UK, a support group (DMD Pathfinders), and 5 UK National Health Service (NHS) Trusts (University College London, Newcastle Upon Tyne, Leeds, University Hospitals Bristol, and Alder Hey). Purposive recruitment was based on a sampling framework to help ensure a breadth of participants’ characteristics across age and disease progression (based on lower limb, upper limb, and respiratory function) and continued until data saturation. Participant characteristics are in Table 1.

**Table 1:** Participant characteristics

Age (years)								
7–9	10–14		15–19		20–29	30–39		≥ 40
2	6		3		4	2		1
Assistance needed to walk								
Walks > 10 min without a mobility device			Walks < 10 min without a mobility device			Always uses a mobility device		
2			5			11		
Assistance needed to transfer (from bed to chair)								
Transfers independently without assistance			Transfers with some support			Needs a person or lifting device to transfer		
6			1			11		
Assistance needed to eat								
Raises hand to mouth with food or drink independently			Raises hand to mouth with food or drink some support			Needs another person to bring food or drink to mouth		
9			2			7		
Assistance needed to breathe								
Does not use a ventilator			Uses a ventilator at night			Uses a ventilator day and night		
14			1			3		
Use of steroid and heart medication								
Uses neither		Uses steroid medication		Uses heart medication			Uses both	
1		3		3			11	

### Procedure

Participants were interviewed in-person or online (to facilitate participants’ preferences) between June and December 2018. Interviews followed a semi-structured topic guide (Online Resource 1), based on a review into HRQoL themes in DMD [5]. The topic guide was reviewed and approved by Duchenne UK Patient and Public Involvement (PPI) representatives and was sent to participants in advance of the interview along with a brief demographics questionnaire and consent form. Written informed consent was obtained from all participants (or participants’ parents in the case of children under 16 years old) prior to the interview. In the case of children, participants and caregivers made a joint decision about whether the caregiver was present during the interview, which happened in 7 cases. Interviews lasted for a mean of 53 min (*SD* = 15). At the start of the interview, participants were exposed to examples of generic, preference-based, PROs used to assess HRQoL in DMD and other conditions (i.e. EQ-5D/EQ-5D-Y [26, 27], Health Utilities Index [28, 29], and/or Child Health Utility-9D [30]) and asked about their impressions of them. During the interview, sensitive issues specific to adults, but not children, such as end of life planning and sexual functioning, were not probed by the interviewer. This decision was taken as the project was focused on identifying commonalities in domains of HRQoL across boys and men with Duchenne, and these topics were considered less appropriate for the former. All interviews were undertaken by a trained qualitative researcher with experience working with and interviewing vulnerable participants. While the interviewer had a background in HRQoL research, they intended not to impose their presuppositions on participants, and allowed space for inductive contributions. No relationship existed between the interviewer and participants prior to the study.

### Analysis

**Fig. 1:**
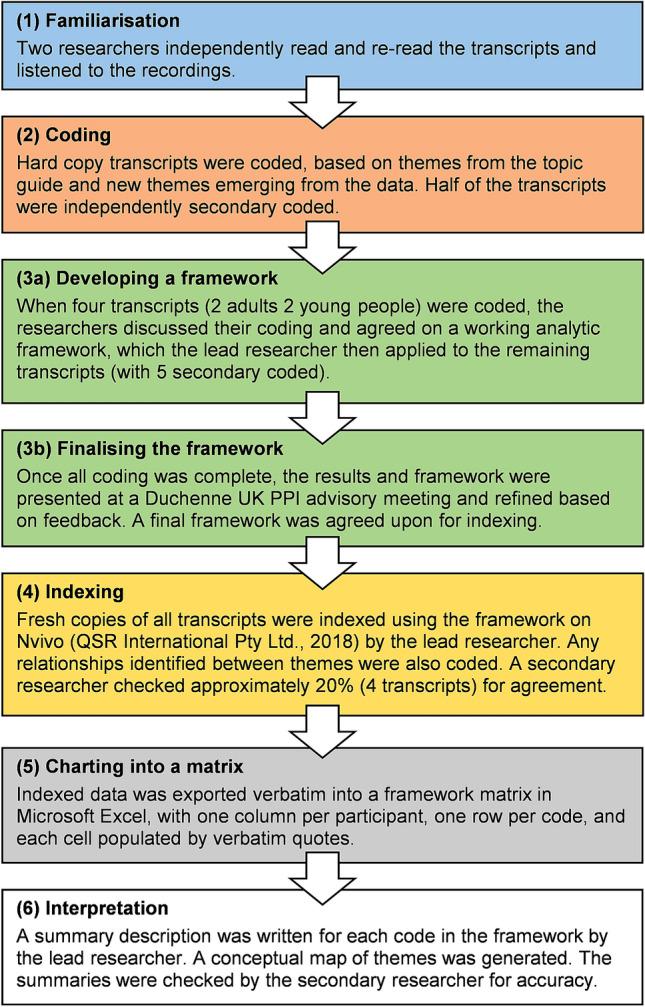
Six stages of framework analysis used in the study, adapted from [32]

Interviews were audio recorded, transcribed verbatim, identifiers removed, and analysed using framework analysis [31], chosen as it facilitates a combination of deductive (based on a priori themes) and inductive (themes from participants) analysis. Framework analysis can be considered independent to a specific philosophical, epistemological, or theoretical paradigm [30]. Analysis was based on 6 iterative stages (adapted from [32]), outlined in Fig. 1. Trustworthiness of the analysis was assured against accepted criteria [33, 34]. In particular, we aimed to enhance the credibility of the analysis through peer debriefing with PPI representatives and researcher triangulation; transferability by providing thick descriptions of the research, data and results; and dependability by documenting all decisions made in the analytic process (i.e. an “audit trail” [33, 34]).

## Results

**Fig. 2:**
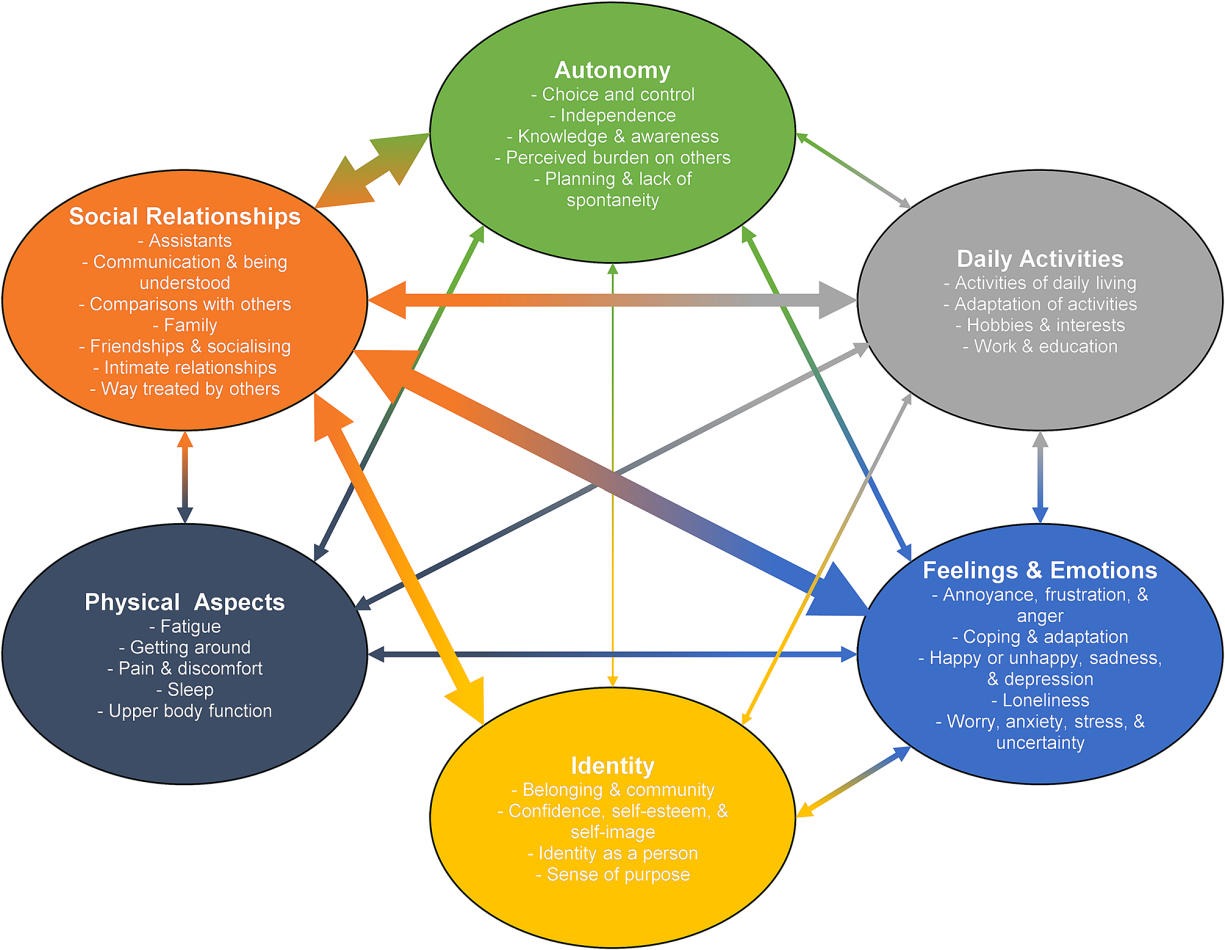
Conceptual mapping between themes in HRQoL framework. Size of arrows represents how often relationship between categories was coded in the qualitative data (after rounding to the nearest 5 and dividing by 5 to approximate a width in pt). Wider arrows represent stronger relationships between categories

Thirty-seven themes were coded, hierarchically organised into seven categories. Six were considered facets of HRQoL and one (healthcare, support, and environment) was considered an input. The framework and data coverage is in Table 2. The relationships between themes are mapped in Fig. 2. An additional three themes were used to code data related to the generic PROs used as stimuli during the interviews. Data saturation was good with no new themes emerging after the first few interviews (Table 3). Results for the six HRQoL themes are discussed below, with results on healthcare, support, and environment and PROs in Online Resource 2. The full indexed, anonymised data supporting this paper is available in Online Resource 3. Participant number is included after each quote, as well as whether they were an adult (20 + years) or young person (7 to 19 years).

**Table 2:** A comprehensive framework for health-related quality of life in Duchenne muscular dystrophy

	Coverage (*N* times coded in data)	
Framework	Participants	References
*Autonomy*		
Choice and control	8	31
Independence	16	80
Knowledge and awareness	5	14
Perceived burden on others	9	24
Planning and lack of spontaneity	7	20
*Daily activities*		
Activities of daily living (e.g. self-care, eating, shopping)	10	26
Adaptation of activities	9	13
Hobbies and interests	12	53
Work and education	17	71
*Feelings and Emotions*		
Annoyance, frustration, and anger	16	41
Coping and adaptation	12	45
Happy or unhappy, sadness, and depression	18	66
Loneliness	6	10
Worry, anxiety, stress, and uncertainty	15	87
*Identity*		
Belonging and community	6	16
Confidence, self-esteem, and self-image	10	57
Identity as a person	9	43
Sense of purpose	5	21
*Physical aspects*		
Fatigue	14	33
Getting around	16	90
Pain and discomfort	14	33
Sleep	9	13
Upper body function	7	8
*Social relationships*		
Assistants	12	63
Communication and being understood	14	36
Comparisons with others	11	35
Family	12	31
Friendships and socialising	18	155
Intimate relationships	8	31
Way treated by others	17	125

*N* = 18. Participants = Number of transcripts in which this theme was coded. References = Number of times this theme was coded across all data. Healthcare, support, and environment and subordinate themes (access to healthcare provision, accessibility, assistive technology, burden of healthcare, devices and equipment, medicines, support from others) not featured in the framework as categorised as an input variable to HRQoL

**Table 3:** Data saturation grid

	Transcript indexed																	
Theme	1	2	3	4	5	6	7	8	9	10	11	12	13	14	15	16	17	18
Activities of daily living	X	X	X		X	X		X	X		X						X	X
Adaptation of activities			X	X		X				X	X	X	X	X			X	
Annoyance, frustration, and anger	X		X	X	X	X	X	X	X	X		X	X	X	X	X	X	X
Assistants	X		X	X	X	X	X	X					X	X	X	X	X	
Belonging and community					X		X	X				X		X	X			
Choice and control	X		X	X		X	X	X						X			X	
Communication and being understood	X		X	X	X	X	X	X		X		X	X	X	X	X		X
Comparisons with others	X		X			X	X	X	X	X		X	X	X		X		
Confidence, self-esteem, and self-image	X			X		X	X	X				X		X		X	X	X
Coping and adaptation	X		X	X	X	X	X	X				X		X	X	X	X	
Family	X		X	X		X		X		X		X	X	X	X		X	X
Fatigue	X		X		X	X		X		X	X	X	X	X	X	X	X	X
Friendships and socialising	X	X	X	X	X	X	X	X	X	X	X	X	X	X	X	X	X	X
Getting around	X	X	X	X	X	X	X		X	X	X	X	X	X		X	X	X
Happy or unhappy, sadness, and depression	X	X	X	X	X	X	X	X	X	X	X	X	X	X	X	X	X	X
Hobbies and interests	X				X	X	X		X		X		X	X	X	X	X	X
Identity as a person	X		X	X			X	X				X		X	X		X	
Independence	X	X	X	X	X	X	X	X	X	X	X	X		X		X	X	X
Intimate relationships	X		X	X			X	X				X		X			X	
Knowledge and awareness	X			X		X										X	X	
Loneliness	X			X				X			X			X	X			
Pain and discomfort	X		X	X	X	X			X	X	X		X	X	X	X	X	X
Perceived burden on others	X		X	X		X	X		X			X				X	X	
Planning and lack of spontaneity			X	X			X							X		X	X	X
Sense of purpose				X			X	X				X				X		
Sleep	X	X	X		X					X	X		X	X	X			
Upper body function	X		X	X			X	X	X					X				
Way treated by others	X		X	X	X	X	X	X	X	X	X	X	X	X	X	X	X	X
Work and education	X		X	X	X	X	X	X	X	X	X	X	X	X	X	X	X	X
Worry, anxiety, stress, and uncertainty	X		X	X	X	X	X	X	X	X	X	X	X	X	X		X	

### Autonomy

#### Choice and control

For adult participants and older young people with DMD, the ability to be in control and make choices about things in day-to-day life, including the healthcare received and assistants employed, was identified as empowering. This is particularly the case as control was stripped away:

“I think it’s quite empowering in that, you know, being able to make decisions on the fundamental or fundamental aspects of my life makes me feel more like an adult rather than, you know, a child who’s cared for kind of thing” (P4, Adult).

#### Independence

Independence was identified as important, with Duchenne potentially limiting it. While many were accepting of having assistance, the need to rely on other people could introduce barriers, such as when socialising and the inability to have time away from people. Paradoxically, some participants outlined how being dependent on others could facilitate independence elsewhere:

“I always need someone to either drive the car or take, take me somewhere, cos I’d need someone all the time (…) So I’m independent in my chair, but I’m not sort of independent in the sort of greater community” (P14, Young Person).

#### Knowledge and awareness

Some participants expressed an importance of having knowledge and awareness about their condition and related aspects, such as healthcare. However, the unpredictable nature of Duchenne coupled with it being a progressive condition was noted as problematic:

“I’ve been quite lucky to be able to avoid a lot of the interventions, but then again a lot of that is down to my knowledge and awareness and kind of vigilance really of knowing when I need to be preventative and knowing like the danger of certain things” (P4, Adult).

#### Perceived burden on others

A number of participants were acutely aware of the potential burden that caring for people with Duchenne put on others, including family members, personal assistants, and friends, and there was sometimes associated guilt associated:

“I probably have got mates that would come into the disabled area with me but I don’t want to be a burden on them, when they want to be with everyone else, like most teenagers” (P12, Young Person).

#### Planning and lack of spontaneity

Adult participants and older young people pointed out the significant day-to-day planning involved in having DMD, which reduced independence, involvement, and restricted spontaneity. One participant reported directly rebelling against this and occasionally taking risks by not planning:

“I suppose that again comes back to spontaneity like they can just go somewhere, which I would have joined if things were different (…) there are barriers like that, so it has affected me in that sense” (P17, Adult).

### Daily activities

#### Activities of daily living

Some participants noted problems doing “normal” daily activities. This included shopping, eating, and self-care (e.g. dressing and washing). This was linked to worry about not having people around to help and deterioration in the future:

“I have some problems doing my usual activities (…) Like getting dressed. Having a bath. Wait, I don’t have a bath. I have a shower” (P9, Young Person).

#### Adaptation of activities

Participants noted how they have adapted, and continue to adapt, their choice of activities or the way they do them. Therefore, while their activities are “usual” to them, they may not be to someone without Duchenne. For one young person this was linked to stigma:

“I think that a lot of people don’t understand that there’s certain ways people can do things like other people can do differently, there’s not always just one way of doing something, there’s hundreds of ways of doing different things” (P12, Young Person).

#### Hobbies and interests

The ability to take part in regular, chosen hobbies that participants found enjoyable was identified as an important component of HRQoL, with physical, social, and psychological benefits:

“Like the sport I play and then I sort of get a bit of confidence from that, knowing that I’m good at what I do and stuff, so.” (P6, Young Person).

#### Work and education

Both younger and older participants noted impacts on work and education. For younger people, the impact was largely during recreational time and physical education. Older young people noted the need for extensions and work taking longer. A few participants liked the academic side of school. For adults, impacts on work included limiting career choices, fatigue, and the need for support:

“I sometimes feel like with jobs that might be that’s a bit hard because I don’t feel like I can work like a full five days a week for like twelve hours or six hours because I would get too tired” (P1, Adult).

### Feelings and emotions

#### Annoyance, frustration, and anger

The majority of participants reported feeling angry, frustrated, or annoyed from time-to-time. This included when not being able to do things they wanted, feelings of unfairness and injustice at having Duchenne, and based on comparisons with, or the way they were treated by, others:

“Erm, but obviously if you lose your independence it’s frustrating, you don’t wanna have to ask somebody to do something that you should be able to do, erm, yourself” (P17, Adult).

#### Coping and adaptation

Many participants expressed a positive mental attitude and a degree of acceptance towards their condition and coping with its challenges. Coping strategies included resilience, humour, focusing on solutions, distraction, and mindfulness:

“I think what I do a lot of, er, kind of positive thinking and mindfulness and kind of like meditation in the morning just to calm myself” (P8, Adult).

#### Happy or unhappy, sadness, and depression

Periods of sadness and upset were common, often when introspecting or reflecting on the condition and its impacts. The degree of sadness reported varied, with some not experiencing much, some experiencing sadness but not depression, and some identifying with depression. Some participants identified happiness itself as being a good indicator of HRQoL:

“It just makes me feel a bit sad (…) because I wish I didn’t have it sometimes” (P5, Young Person).

#### Loneliness

Participants talked about loneliness and isolation in different ways. Some reported that they were less likely to be lonely, because they always had people helping them. Others revealed loneliness at being the only person who was disabled in particular contexts:

“It’s kind of the loneliness as well I mean cause once you have most of your friends they’ll be going out, going around doing everything being outside at late night, getting drunk, and then going to parties and it feels like you kind of feel a bit left out” (P1, Adult).

#### Worry, anxiety, stress, and uncertainty

Regarding emotional experiences related to Duchenne, worry and anxiety were the most common. These feelings tended to arise around involvement in daily and/or social activities, around health, dependency on others, and worry about the future:

“I mean you can get a bit anxious as well. Because, obviously with having care you have to rely on people so you have to be able to trust that they can do what you need, but if somebody isn’t like that (…) like you do get a bit anxious and worried about things” (P3, Adult).

### Identity

#### Belonging and community

Having a sense of community and belonging was important to participants. This included communal events and activities, where participants felt similar to others and not judged:

“Everyone is accepting of everyone else. All sorts of body shapes, dressing up in all sorts of costumes and everybody just gets on, no judgement, kids come up to you want your photo.” (P7, Adult).

#### Confidence, self-esteem, and self-image

Having confidence, for example to engage with others socially, was associated with a better HRQoL. Confidence and self-esteem fluctuated over time and place. Participants expressed concern about their self-image and the way they appeared to others. For example, concerns about weight (e.g. as a side-effect of steroids) were shared. A couple of participants identified positive effects on self-esteem, developed through a sense of self-love, with Duchenne making them stronger and more determined:

“I sometimes feel like I’m less attractive, like compared to other men because they would get the attention whereas me like I kind of don’t get as much as other people.” (P1, Adult).

#### Identity as a person

While having their own personal identity was important, many older participants wanted to be treated like any “normal” adult. Participants did not want to be defined by Duchenne and wanted people to see beyond their condition:

“Well the thing is like, err, I’d wanna be seen like everyone else, I just. My physical body’s different, but I’m kind of identity isn’t just tied into physical. So you, you just wanna be acknowledged.” (P8, Adult).

#### Sense of purpose

Having a sense of purpose and contributing to society was identified as important for some participants. This included a personal sense of fulfilment for doing things that helped others (e.g. charity work):

“I go to meetings with my local mental health charity and help train their staff telephones to process. And feeling worthwhile as well, being able to do something, even if you can’t work, you’re able to do something that’s worthwhile, helping others.” (P7, Adult).

### Physical aspects

#### Fatigue

Participants commonly reported experiencing fatigue and tiredness, but the degree of fatigue experienced differed. Tiredness was noted after periods of physical activity, for younger children this included running, but for older participants this included less pronounced activity (e.g. holding a drink):

“Every time I run I get tired.” (P11, Young Person).

#### Getting around

Mobility was described as much more than being able to walk and instead was characterised by adults as the ability to “get around”. Further, losing the ability to walk was not related to a linear decline in HRQoL, but transitioning to a wheelchair had benefits for younger people who may be struggling to walk (e.g. increased mobility, less fear of falling). Some participants spoke about the wellbeing benefits of getting outside and being in the world:

“It’s too focused on walking err, which yeah just seems to be kind of like, in a way, the be all and end all, like you’ve got all these possible options of erm kind of able to walk a little bit and then suddenly not able to walk at all and it’s like (…) there’s so many distinctions within the walking category and no distinction in the non-walking category, which isn’t actually kind of representative of mobility, particularly not with a condition like this, which is so progressive.” (P4, Adult).

#### Pain and discomfort

Participants noted incidences of pain and discomfort that occurred in response to particular triggers and were more pronounced as participants got older. These included feelings of stiffness as a result of being stuck in particular positions, in response to knocks (e.g. while travelling), and the need to do daily physio:

“Yeah, because it all like, it always hurts when I do the stretches. I mean it’s only when they do the stretches where they have to move my knees or push my knee towards my chest.” (P9, Young Person).

#### Sleep

Participants differed in the extent to which they identified their sleep being disrupted. Most younger people stated their sleep was unaffected, but one participant struggled to sleep because they felt worried and upset. Adult participants identified discomfort or ventilation as an impediment to sleep and also acknowledged the impact on others (e.g. having to get up to roll them over in the night):

“I couldn’t get to sleep cause I was in too much pain.” (P5, Young Person).

#### Upper body function

Adult participants and older young people described how their upper body and fine motor function was affected, experiencing a gradual deterioration in functionality over time:

“Your arms as well, a little bit gradually as well, just. But over time they just get, cos I can still lift my arms to only here really. So, yeah, it’s just very gradual, gradually gets weaker over time.” (P14, Young Person).

### Social relationships

#### Assistants

The relationship that participants had with their (personal) assistants was very important for their day-to-day HRQoL, both on an interpersonal level, but also in terms of trust and perceived competency:

“Yeah so you have to have some form of relationship where you can, yeah cause you’ve got to be able to deal with it and you’ve also got to be able to get on with them otherwise it doesn’t work.” (P3, Adult).

#### Communication and being understood

For some adults, such as those on ventilation, difficulties with speech and being understood by others was a concern. Having the confidence to talk to others was hampered for some participants but not others:

“One of the ones that is relevant, is at the top is actually being understood, erm. So, err, particularly for me like since using the ventilator, erm, again I just, I suppose I would have to tick able to understood partially” (P4, Adult).

#### Comparisons with others

Participants frequently made comparisons to peers without Duchenne and this had an impact on their subjective HRQoL. For example, for adults and older young people, peers in intimate relationships, or for younger people, peers running about. Alternatively, some participants made comparisons with other disabled people and saw themselves as better off:

“They make me feel like, erm, other people are doing stuff like slightly better than me. Like say if we’re in a race like some people just go like ever so slowly past me.” (P10, Young Person).

#### Family

Participants’ family were noted as an important source of support. However, many participants talked about the need for family members to have some time to be family members and not just caregivers. Some participants mentioned a degree of guilt over this:

“But I could have done with possibly having a bit of rest, a break from my family in a way but err cause it sort of means (…) basically everybody wants to have a break.” (P6, Young Person).

#### Friendships and socialising

As the most coded theme, friendships and socialising were noted as a hugely important component of HRQoL. Many adults and older young people acknowledged that the condition had affected their friendships and ability to socialise. Friendships with similar “disabled” others were viewed as positive in some regards, such as having a shared experience, but some participants felt their friendships had been artificially restricted to people with disabilities. Being included socially was very important for most participants:

“You sort of want to just be with a certain group of friends but sometimes when it gets, almost feels like it’s been forced on you that you’ve got to spend time with, I don’t know, a certain person and sort of only having the option of, I don’t know, sitting and throwing a ball” (P6, Young Person).

#### Intimate relationships

For adults and older young people, having a partner or an intimate relationship was seen as relevant to overall HRQoL, although the degree of importance was variable. Participants recognised that Duchenne could be a barrier to developing relationships and there was stigma:

“I’ve met a lot of boys with Duchene that kind of they’ve got a girlfriend, and some are married, and one of them, kind of he has a child. So that without that, I think intimate relationships shouldn’t be kind of ignored, because usually for disabled people, you know, it is, is, isn’t an area that’s spoken about.” (P8, Adult).

#### Way treated by others

The way other people treated participants was the second most coded theme. Adult and older young people talked about the negative effects of being treated differently, including others talking to an assistant rather than them, pre-judging, or infantilising them. Younger participants were generally less aware of this, but some instances of teasing and bullying were disclosed:

“Like talking to me as if I’m really young and have difficulty understanding speech, when it’s not fair to assume that.” (P15, Young Person).

## Discussion

While HRQoL has been assessed quantitatively, no qualitative evidence has been published fully detailing the lived experience and breadth of HRQoL in people with Duchenne. We present here a comprehensive framework for characterising HRQoL in DMD. The research is broadly consistent with what is known from prior studies, detailing, for example, experiences of fatigue, pain, and anxiety [35]; limits on independence [36]; and impacted social activities [37], but provides additional breadth and depth. The work builds on a previous HRQoL review and highlights quality of life domains linked to health that may have featured less often in the literature including: confidence and self-esteem; identity as a person; annoyance, frustration, and anger; belonging and community; sense of purpose; loneliness; planning and lack of spontaneity; relationships with (personal) assistants; and more [5].

Most work on HRQoL in DMD has focused on how it differs between samples, across time, or in response to interventions [5], rather than exploring the relative importance of different aspects of HRQoL for people with Duchenne. Notwithstanding that coverage is often an imperfect indicator of importance, the most coded themes were around social relationships (i.e. friendships and socialising and way treated by others). This was also a domain that participants reported being inadequately covered in the generic preference-based PROs used in the interviews. Together, this suggests that participants placed importance on social aspects of HRQoL. Social participation has been shown to be restricted in prior research [38–40] and may be a meaningful outcome target for future interventions to improve HRQoL in Duchenne. Reinforcing their importance, social relationships are a key component of many non-preference-based HRQoL measures, such as the Pediatric Quality of Life Inventory [41], and thus their omission in popular preference-based PROs, used for cost-effectiveness analysis, is potentially problematic for multiple health conditions, including DMD.

In addition to the omission of social items, participants were critical of other aspects of the preference-based PROs shown to them, including the EQ-5D. This included a restricted definition of mobility and vagueness around “usual activities” (Online Resource 2). While preliminary, these insights suggest that while scales such as the EQ-5D may perform well psychometrically in Duchenne [42] they may suffer from a critical lack of content validity [43]. The consequence of this is that Duchenne HRQoL data (e.g. from the EQ-5D) being used in cost-effectiveness analysis may not fully reflect any changes (or lack thereof) in elements of health-related HRQoL that matter to the people designed to benefit from health technologies, and this should form the basis for future research and debate [43, 44].

Potential limitations of the work include selection bias; participants were self-selected volunteers and while a breadth of people were recruited, “hidden” voices and those harder-to-reach participants living with Duchenne may not be adequately represented. Second, it is difficult to assess the degree of transferability of the findings to other countries and cultural groups, and future research could explore this with purposive sampling in different contexts. Third, allowing the presence of caregivers when interviewing children is ethically correct, but may have influenced their responses in the interviews. Fourth, adult-specific issues, such as sexual functioning, were not covered in the interviews, yet have been shown to be important issues for adults with Duchenne [17, 45, 46]. Future work could explore the degree to whether these, and related components, are critical omissions in the proposed HRQoL framework for adults with DMD.

In conclusion, a 30-item framework for HRQoL in DMD has been developed, which was used as input into the development of a new condition-specific HRQoL PRO: the DMD-QoL [24, 25]. This data has rich value in its own right in helping to showcase the lived experience and HRQoL of people living with Duchenne and act as a barometer for which to assess tools used to quantify HRQoL in people with DMD. Beyond this, the findings have implications for broader HRQoL measurement using preference-based PROs that are used in cost-effectiveness analyses, highlighting the potentially problematic omission of social items and a potential lack of content validity in certain health conditions that should be further investigated.

## Supplementary Information

Below is the link to the electronic supplementary material.

Supplementary file1 (PDF 655 kb)

Supplementary file2 (PDF 611 kb)

Supplementary file3 (XLSX 199 kb)

## References

[1] Crisafulli S, Sultana J, Fontana A, Salvo F, Messina S, Trifirò G (2020). Global epidemiology of Duchenne muscular dystrophy: An updated systematic review and meta-analysis. Orphanet Journal of Rare Diseases.

[2] Landfeldt E, Thompson R, Sejersen T, McMillan HJ, Kirschner J, Lochmüller H (2020). Life expectancy at birth in Duchenne muscular dystrophy: A systematic review and meta-analysis. European Journal of Epidemiology.

[3] Mayo NE (2015). ISOQOL dictionary of quality of life and health outcomes measurement.

[4] Powell PA, Carlton J, Woods HB, Mazzone P (2020). Measuring quality of life in Duchenne muscular dystrophy: A systematic review of the content and structural validity of commonly used instruments. Health and Quality of Life Outcomes.

[5] Uttley L, Carlton J, Woods HB, Brazier J (2018). A review of quality of life themes in Duchenne muscular dystrophy for patients and carers. Health and Quality of Life Outcomes.

[6] Theadom A, Rodrigues M, Ranta A, Poke G, Love D, Jones K, Ao BT, Hammond-Tooke G, Parmar P, O’Grady G (2021). Impact and predictors of quality of life in adults diagnosed with a genetic muscle disorder: A nationwide population-based study. Quality of Life Research.

[7] Szabo SM, Audhya IF, Malone DC, Feeny D, Gooch KL (2020). Characterizing health state utilities associated with Duchenne muscular dystrophy: A systematic review. Quality of Life Research.

[8] Bendixen RM, Morgenroth LP, Clinard KL (2016). Engaging participants in rare disease research: a qualitative study of Duchenne muscular dystrophy. Clinical Therapeutics.

[9] Skyrme S (2017). In and on their own terms: Children and young people's accounts of life with Duchenne muscular dystrophy. Child Care in Practice.

[10] Landrum Peay H, Fischer R, Tzeng JP, Hesterlee SE, Morris C, Strong Martin A, Rensch C, Smith E, Ricotti V, Beaverson K (2019). Gene therapy as a potential therapeutic option for Duchenne muscular dystrophy: A qualitative preference study of patients and parents. PLoS ONE.

[11] Dreyer PS, Steffensen BF, Pedersen BD (2010). Living with severe physical impairment, Duchenne’s muscular dystrophy and home mechanical ventilation. International Journal of Qualitative Studies on Health and Well-Being.

[12] Walker M, Mistry B, Amin R, McAdam L, Kalnins D, Lui T, McPherson AC (2021). A qualitative exploration of the priorities and experiences of children with Duchenne muscular dystrophy, their parents, and healthcare professionals around weight management. Disability and Rehabilitation.

[13] Gibson BE, Young NL, Upshur RE, McKeever P (2007). Men on the margin: A Bourdieusian examination of living into adulthood with muscular dystrophy. Social Science & Medicine.

[14] Gibson BE, Zitzelsberger H, McKeever P (2009). ‘Futureless persons’: Shifting life expectancies and the vicissitudes of progressive illness. Sociology of Health & Illness.

[15] Gibson BE, Mistry B, Smith B, Yoshida KK, Abbott D, Lindsay S, Hamdani Y (2014). Becoming men: Gender, disability, and transitioning to adulthood. Health.

[16] Read J, Kinali M, Muntoni F, Weaver T, Garralda ME (2011). Siblings of young people with Duchenne muscular dystrophy–a qualitative study of impact and coping. European Journal of Paediatric Neurology.

[17] Abbott D, Prescott H, Forbes K, Fraser J, Majumdar A (2017). Men with Duchenne muscular dystrophy and end of life planning. Neuromuscular Disorders.

[18] Pehler S-R, Craft-Rosenberg M (2009). Longing: The lived experience of spirituality in adolescents with Duchenne muscular dystrophy. Journal of Pediatric Nursing.

[19] Abbott D, Jepson M, Hastie J (2016). Men living with long-term conditions: Exploring gender and improving social care. Health & Social Care in the Community.

[20] Yamaguchi M, Suzuki M (2013). Independent living with Duchenne muscular dystrophy and home mechanical ventilation in areas of Japan with insufficient national welfare services. International Journal of Qualitative Studies on Health and Well-Being.

[21] Finkelstein A, Marcus E-L (2018). Realizing autonomy: The phenomenology of independence and interdependence while living with Duchenne muscular dystrophy. Disability & Society.

[22] Donnelly CM, Quinlivan RM, Herron A, Graham CD (2022). A systematic review and qualitative synthesis of the experiences of parents of individuals living with Duchenne muscular dystrophy. Disability and Rehabilitation.

[23] Powell PA, Carlton J, Rowen D, Brazier JE (2019). Producing a preference-based quality of life measure for people with Duchenne muscular dystrophy: A mixed-methods study protocol. British Medical Journal Open.

[24] Powell PA, Carlton J, Rowen D, Chandler F, Guglieri M, Brazier JE (2021). Development of a new quality of life measure for Duchenne muscular dystrophy using mixed methods: The DMD-QoL. Neurology.

[25] Rowen D, Powell P, Mukuria C, Carlton J, Norman R, Brazier J (2021). Deriving a preference-based measure for people with Duchenne muscular dystrophy from the DMD-QoL. Value in Health.

[26] Rabin R, Charro F (2001). EQ-SD: A measure of health status from the EuroQol Group. Annals of Medicine.

[27] Wille N, Badia X, Bonsel G, Burström K, Cavrini G, Devlin N, Egmar A-C, Greiner W, Gusi N, Herdman M (2010). Development of the EQ-5D-Y: A child-friendly version of the EQ-5D. Quality of Life Research.

[28] Feeny D, Furlong W, Boyle M, Torrance GW (1995). Multi-attribute health status classification systems. Pharmaco Economics.

[29] Torrance GW, Feeny DH, Furlong WJ, Barr RD, Zhang Y, Wang Q (1996). Multiattribute utility function for a comprehensive health status classification system: Health utilities index mark 2. Medical Care.

[30] Stevens K (2009). Developing a descriptive system for a new preference-based measure of health-related quality of life for children. Quality of Life Research.

[31] Ritchie J, Spencer L, Bryman A, Burgess R (1994). Qualitative data analysis for applied policy research. Analysing Qualitative Data.

[32] Gale NK, Heath G, Cameron E, Rashid S, Redwood S (2013). Using the framework method for the analysis of qualitative data in multi-disciplinary health research. BMC Medical Research Methodology.

[33] Lincoln, Y. S., & Guba, E. G. (1985) Naturalistic inquiry: sage

[34] Guba, E. G., & Lincoln, Y. S. (1989) Fourth generation evaluation: Sage

[35] Pangalila RF, van den Bos GA, Bartels B, Bergen M, Stam HJ, Roebroeck ME (2015). Prevalence of fatigue, pain, and affective disorders in adults with duchenne muscular dystrophy and their associations with quality of life. Archives of Physical Medicine and Rehabilitation.

[36] Madsen A, Rahbek J, Werge B, Marquardt J, Gredal O, Steffensen B (2014). GP 304: Living conditions and quality of life in adults with Duchenne muscular dystrophy–A Danish survey. Neuromuscular Disorders.

[37] Elsenbruch S, Schmid J, Lutz S, Geers B, Schara U (2013). Self-reported quality of life and depressive symptoms in children, adolescents, and adults with Duchenne muscular dystrophy: A cross-sectional survey study. Neuropediatrics.

[38] Bendixen RM, Senesac C, Lott DJ, Vandenborne K (2012). Participation and quality of life in children with Duchenne muscular dystrophy using the international classification of functioning, disability, and health. Health and Quality of Life Outcomes.

[39] Houwen-van Opstal SLS, Heutinck L, Jansen M, Krom YD, Cup EHC, Hendriksen JGM, Willemsen MAAP, Verschuuren JJGM, Niks EH, de Groot IJM (2021). Occurrence of symptoms in different stages of Duchenne muscular dystrophy and their impact on social participation. Muscle & Nerve.

[40] Abbott D, Carpenter J (2015). “The things that are inside of you are horrible”: children and young men with duchenne muscular dystrophy talk about the impact of living with a long-term condition. Child Care in Practice.

[41] Varni JW, Seid M, Rode CA (1999). The PedsQL™: Measurement model for the pediatric quality of life inventory. Medical Care.

[42] Crossnohere NL, Fischer R, Lloyd A, Prosser LA, Bridges JFP (2021). Assessing the appropriateness of the EQ-5D for duchenne muscular dystrophy: a patient-centered study. Medical Decision Making.

[43] Powell PA, Carlton J, Rowen D, Brazier J, Facey K, Bayley K, Chandler F, Godfrey J, Crossley E (2021). Measuring what matters: little evidence supporting the content validity of EQ-5D in people with duchenne muscular dystrophy and their caregivers. Medical Decision Making.

[44] Crossnohere NL, Fischer R, Lloyd A, Prosser LA, Bridges JFP (2022). Authors’ response to comment on “assessing the appropriateness of the EQ-5D for duchenne muscular dystrophy: a patient-centered study”. Medical Decision Making.

[45] Abbott D, Carpenter J, Gibson BE, Hastie J, Jepson M, Smith B (2019). Disabled men with muscular dystrophy negotiate gender. Disability & Society.

[46] Abbott D, Carpenter J (2014). ‘Wasting precious time’: Young men with Duchenne muscular dystrophy negotiate the transition to adulthood. Disability & Society.

