# Supplementary material for: A comprehensive qualitative framework for health-related quality of life in Duchenne muscular dystrophy
Source: Qual Life Res. 2022 Sep 1;32(1):225–36. doi: 10.1007/s11136-022-03240-w (PMC9829595; doi:10.1007/s11136-022-03240-w)
Supplement: Supplementary file 1 — Supplementary file1 (PDF 655 kb) [file 11136_2022_3240_MOESM1_ESM.pdf]

Article Title: A Comprehensive Qualitative Framework for Health-Related Quality of Life in Duchenne Muscular Dystrophy

Journal Name: Quality of Life Research

Authors: Philip A. Powell, Jill Carlton

Corresponding Author: Philip A. Powell, University of Sheffield, UK, p.a.powell@sheffield.ac.uk

Online Resource 1 – Interview Topic Guide

BETTER MEASURING QUALITY OF LIFE IN PEOPLE WITH DMD

Interview Schedule

Before the interview:

- The interviewer will introduce themselves, and thank you for agreeing to take part.

- They will provide some background to this research:

  "This study is funded by the charity Duchenne UK and is being carried out by the University of Sheffield. The aim of the study is to explore and understand the things that are important in determining quality of life (QoL) in people with Duchenne Muscular Dystrophy (DMD). By knowing how and in which ways DMD affects people's QoL, people who make health care decisions and doctors will know what to measure when they are testing any new treatments designed to improve QoL. While we may touch on things that may have affected you in the past, or may affect you in the future, we are interested in the things that are important to you today, in the present."

- The interviewer will go through some important points with you:
  - With your permission we would like to record this interview. This will help us to focus on the things you say.
  - Everything you say is confidential. Information you share in this interview will not be passed on to others outside of the research team, except if it is information that suggests you may be a threat to yourself or others, which we have a duty to report. Any quotes used in reports will be anonymised.
  - In the interview, we might discuss things you may find difficult. You do not have to answer any questions you do not want to. If there are any questions that you are uncomfortable with, or you find difficult, we can move on, so please don't worry.
  - The interview will take us approximately an hour to complete. However, you can stop the interview at any time, or take a break, if you wish to do so.
  - We would like to conduct the interview with you personally to hear all of your own views on the issues we discuss. However, it is up to you whether you take part in the interview alone, or with someone else present. We want to feel comfortable and for the interview to fit-in practically with your daily life. If you do decide to have someone with you in the interview, they will not be able to take part in the interview directly, as we are interested in your personal views.

- The interviewer will provide a further copy of the information sheet, and, if you have not done so already, ask you to complete a consent form and for you to complete a form with some background details about you.

- The interviewer will ask you if there is anything you would like to ask us before we begin.

Questions to cover during the interview:

1. I'd like to start by showing you some questions that are used to assess people's quality of life and make decisions on the effectiveness of treatments [*Refer to questions from copies of the EQ-5D, EQ-5D-Y, HUI2, and CHU-9D]*. Can you please have a look at these questions for me and tell me whether you think any topics are missing that would be important to assess your quality of life in relation to Duchenne Muscular Dystrophy (DMD). Please let me know at any time if you would like any help reading or understanding the questions.

2. Okay, thanks, I'd now like to ask you about several areas of your life in turn, to see in each area which aspects are important to you personally. Let's start by talking about the physical factors that may affect your quality of life. What impacts on your physical functioning does DMD have?

   [*prompts: physical mobility and getting around; effects of sedentary behaviour on physical health; difficulties with sleep; frequency, intensity, and occurrence of pain; dexterity; problems with usual activities or those of day-to-day living*]

   [*Refer to the copies of the EQ-5D, EQ-5D-Y, HUI2, and CHU-9D*]. Do you think these measures adequately capture these things you have mentioned?

3. Thank you. Let's move on now to talk about your psychological health. In what ways does living with DMD affect your mental health, or the way you feel emotionally and/or psychologically?

   [*prompts: does it affect your happiness or mood?; do you feel depressed?; do you feel anxious?; are you able to cope with DMD?, what coping strategies do you have? Do you have any difficulties in communicating with others, or particular people?*]

   [*Refer to the copies of the EQ-5D, EQ-5D-Y, HUI2, and CHU-9D*]. Do you think these measures adequately capture these things you have mentioned?

4. Thanks. Let's move on to talk about the social aspects of your life. In what ways does having DMD affect your ability to socialise and take part in things with others?

   [*prompts: participation in social and leisure activities; friends and social networks; intimate relationships with others; effects on studying or work*]

   [*Refer to the copies of the EQ-5D, EQ-5D-Y, HUI2, and CHU-9D*]. Do you think these measures adequately capture these things you have mentioned?

5. Okay, thank you. Can we talk now about your sense of identity and independence? What effects do you think DMD has on your independence and the way you are treated by others?

   [*prompts: dependence on others and self-care; autonomy; dignity and respect from others; fatigue*]

   [*Refer to the copies of the EQ-5D, EQ-5D-Y, HUI2, and CHU-9D*]. Do you think these measures adequately capture these things you have mentioned?

6. Thank you. Can I now ask about your use of healthcare services and equipment? Are there any devices, equipment, medications, and/or other treatments for DMD that have affected your quality of life in a good or bad way?
   [*prompts: accessibility/wheelchair use; healthcare service provision of equipment and devices; treatment related/therapy effects, including medications (e.g. for pain)*]

   [*Refer to the copies of the EQ-5D, EQ-5D-Y, HUI2, and CHU-9D*]. Do you think these measures adequately capture these things you have mentioned?

7. Thank you very much. Aside from the topics we have covered so far, are there any other ways that DMD affects your quality of life? This could be anything that is important to you. Please share as much information as you are able.

Close:
The interviewer will turn off the recording and ask you if you have any questions about the project. They will explain the next stages of the project, and ask for your consent to be contacted again. They will then thank you for your time.

***

BETTER MEASURING QUALITY OF LIFE IN PEOPLE WITH DMD

Interview Schedule (Under 16s)

Before the interview:

- The interviewer will introduce themselves, and thank you and your child for agreeing to take part.

- They will provide some background to this research:

  "This study is funded by the charity Duchenne UK and is being carried out by the University of Sheffield. The study will explore and understand the things that are important for quality of life (QoL) in people with Duchenne Muscular Dystrophy (DMD). By knowing how and in what ways DMD affects people's QoL, doctors will know what to measure when they are testing new treatments designed to improve QoL. While we may touch on things that may have affected you in the past, or may affect you in the future, we are interested in the things that are important to you today, in the present."

- The interviewer will go through some important points with you and your child:
  - With your permission we would like to record this interview. This will help us to focus on the things you say.

- Everything you say is private, unless it is something that suggests you are in danger and then we have to report it to keep you safe. While quotes from this interview may be used in a report, these will not be linked to your name.
- In the interview, we might discuss things that you may find hard. You do not have to answer any questions that you do not want to. If there are any questions that you are uncomfortable with, or you find hard, we can move on, so please don't worry.
- The interview will take us about an hour to complete. However, you can stop the interview at any time, or take a break, if you want to.
- We would like to hear all of your own views. It is up to you both whether you (the child) take part in the interview alone or with someone else with you. We want you to feel comfortable and for the interview to fit-in with your life. If you do decide to have someone with you, they will not be able to answer the questions themselves, as we are interested in your views. If your parent or guardian is not with you in the interview itself, we would like to ask them to stay nearby, in case you need them. Please let us know at any time if you do.

Questions to cover during the interview:

1. I'd like to start by showing you some questions that are designed to work out how Duchenne Muscular Dystrophy (DMD) affects your life [*Refer to questions from copies of the EQ-5D-Y, HUI2, and CHU-9D*]. Can you please have a look at these questions for me and tell me whether you think there is anything missing that is important to you? Please let me know at anything time if you would like any help reading or understanding the questions.

2. Okay, thanks, I'd now like to ask you about several different bits of your life, to see which aspects are important to you.

   Let's start by talking about the physical bits of having DMD. What effects does DMD have on you doing things with your body and your daily physical activities?

   [*prompts: walking and getting around; effects of not being able to get around on physical health; difficulty sleeping; having pain; doing things with your hands; problems with usual activities or those of day-to-day living*]

   [*Refer to the copies of the EQ-5D-Y, HUI2, CHU-9D*]. Do you think these questions ask about what you have just mentioned?

3. Thank you. Let's move on now to talk about how DMD makes you feel. How does having DMD make you feel?

   [*prompts: does it make you feel sad?; do you feel worried?; do you feel like you can cope with DMD?; what do you do to cope with it?; do you find it hard to talk to others, or some people?]*

   [*Refer to the copies of the EQ-5D-Y, HUI2, CHU-9D*]. Do you think these questions ask about what you have just mentioned?

4. Thanks. Let's move on to talk about doing fun things and your friends. Does having DMD affect the things you can do for fun and with your friends and others?
   [*prompts: taking part in trips out and games; friends and groups; having a boyfriend or girlfriend (if applicable); effects on school and school work*]

   [*Refer to the copies of the EQ-5D-Y, HUI2, CHU-9D*]. Do you think these questions ask about what you have just mentioned?

5. Okay, thank you. Can we talk now about how you feel about yourself and doing things by yourself? How does DMD affect these things?
   [*prompts: do you have to ask other people to do things for you a lot?; what about washing and dressing?; do you feel like you can do things yourself?; do other people treat you any differently?; do you get tired?*]

   [*Refer to the copies of the EQ-5D-Y, HUI2, CHU-9D*]. Do you think these questions ask about what you have just mentioned?

6. Thank you. Let's talk about the things you use to help you. Are there any things you use to help you do things or feel better day-to-day, and are they good or bad?
   [*prompts: do you use a stick or wheelchair?; do you use any equipment or things to help you feel better? do you have to take any medicines (e.g. for pain)?*]

   [*Refer to the copies of the EQ-5D-Y, HUI2, CHU-9D*]. Do you think these questions ask about what you have just mentioned?

7. Thank you so much. As well as what we have talked about, are there any other ways that DMD affects your life? This could be anything that is important to you. Please tell us as much as you can, it is really helpful.

Close:
The interviewer will turn off the recording and ask you if you have any questions about what the interview is about. They will tell you what is happening next, and ask for your parent or guardian's consent to contact you again if you want to take part in some more research. They will then thank you for talking to him.
