# Supplementary material for: A comprehensive qualitative framework for health-related quality of life in Duchenne muscular dystrophy
Source: Qual Life Res. 2022 Sep 1;32(1):225–36. doi: 10.1007/s11136-022-03240-w (PMC9829595; doi:10.1007/s11136-022-03240-w)
Supplement: Supplementary file 2 — Supplementary file2 (PDF 611 kb) [file 11136_2022_3240_MOESM2_ESM.pdf]

Article Title: A Comprehensive Qualitative Framework for Health-Related Quality of Life in Duchenne Muscular Dystrophy

Journal Name: Quality of Life Research

Authors: Philip A. Powell, Jill Carlton

Corresponding Author: Philip A. Powell, University of Sheffield, UK, p.a.powell@sheffield.ac.uk

Online Resource 2 – Supplemental Results

Healthcare, Support, and Environment

Access to healthcare provision

Many participants expressed barriers to accessing healthcare provisions, such as high-quality equipment, which was detrimental to their quality of life (QoL). There was a critical need for the right kind of equipment, such as a high quality chair, that made a big difference to participants' everyday life. Barriers to accessing equipment were largely financial, with an inequity in access driven by means:

"Being able to choose like the wheelchair I want is really, really important. I mean that meant I had to purchase it privately so erm, you know, having that choice and control is dependent on having money" (P4, Adult).

Accessibility

Participants noted how buildings, physical spaces, and services like transport not being accessible affected their lives. A lack of accessibility impacted on participants ability and desire to go to places, do particular activities, and socialise with others (e.g., not being able to shop, go to concerts, or to go and play/socialise at other people's houses or at certain venues). There was additional planning involved in having to check whether places were accessible before travelling to them (and whether they had the necessary equipment to make the facilities accessible). Accessibility as a barrier restricted places people could go and even the choices they made in life (e.g., choosing to go to a campus University as it was more accessible):

"It's alright but some places aren't thought about people in wheelchairs. They don't really think about what they can improve. I quite like shopping but they sometimes have steps that you can't get in." (P2, Young Person).

Assistive technology

A few adult participants mentioned assistive technology as an important vehicle for enabling a greater fulfilment of QoL via increased participation socially and with technology. Assistive technology included that more specialised than standard devices and equipment and could be adapted to be used by all people with Duchenne at different stages of the condition:

"Yeah, I mean, erm things like using the computer and those questionnaires they mention like not using hands and things, but I can still use my mouth, communicate that way, on-screen keyboard, you know, that kind of thing should be in there, shouldn't just be, you can't do anything if you can't use the rest of your body." (P7, Adult).

Burden of healthcare

A few participants alluded to the burden of the amount of healthcare they received and the impact of this on their QoL (e.g. having to go to the hospital a lot). The extent to which participants reported that this affected them was variable:

"I think with, with Duchenne, erm, like the more kind of health and medicalised interventions you need the lower your quality of life will get because you will get more and more issues associated with them (…) if it all goes well then that can enhance your quality of life, but often, you know, there's all sorts of issues you can have with those and they all compound on each, on each other and yeah you basically just end up spending most of your time either wi, in hospital or kind of having appointments and visits or worrying about your health and kind of not being able to get out and about so it can get into a quite difficult position." (P4, Adult).

Devices and equipment

Devices and equipment were essential in supporting the quality of life of people with Duchenne, with the quality of that equipment being a major determinant of outcomes, such as having an appropriate wheelchair that facilitated participants' whole body mobility, above that of a basic chair. On the whole, equipment was noted as an enabler, allowing participants, for example, increased means of mobility to do the things they wanted to do. However, a few participants identified devices and equipment as a barrier to social participation, such as the intrusiveness of ventilator equipment and/or a power chair:

"Yeah, cos the sort of first chair you get from the NHS and things are not always the best and they're not always the most suited for you, cos it's just one they sort of they've found around for you for you to just use

on like a loan. But then as you get, cos this is a privately funded chair, so as you sort of get older your needs get more and you need more support and things in your chair, cos then it starts, your back starts to curve, which mine did and I had to have, had to have this. But, yeah, you need, you start needing more support on the sides and things and your hips and all that, so that's when these chairs are really important for that." (P14, Young Person).

Medicines

While participants that were taking medicines understood the benefits that they provided, concern was raised about certain side-effects of these and their impact on QoL. This included, for example, the effects of steroids on weight gain and facial appearance:

"I get effects from them. Like tiredness and putting on weight (…) And also I'm at a risk of getting cataracts in my eyes from taking these." (P18, Young Person).

Support from others

Participants mentioned the importance of a wide variety of support from others in supporting their day-to-day QoL, including healthcare professionals and services or communities providing knowledge and information (e.g. about accessible places to visit):

"Sometimes you might find something difficult and then they might say actually did you know if you look up this organisation or did you know actually that thing you thought wasn't possible actually is cos I've, I've done it, or. So there's that sharing of sort of information like that, something useful." (P16, Adult).

Patient Reported Outcomes

CHU-9D

In general, the CHU-9D was received well by young participants, who felt able to understand and complete the questionnaire. Only a couple of aspects were identified as potentially missing by a couple of participants, including stress and more focus on socialising:

"Participant: Well I think they might need to. I think again. I think the social aspect should be improved in them.

Interviewer: Okay. Cos the closest there is potentially around joining in.

Participant: I wouldn't really consider that." (P15, Young Person).

EQ-5D

Participants received the EQ-5D measures relatively critically, particularly older participants including older young people and adults. While EQ-5D was noted as easy to read and understand, problems were raised with some of the items in the measure. In particular, the mobility item was criticised as not being applicable or relevant to people with Duchenne who were no longer walking. The questionnaire was criticised for being too focused on walking and that there were other forms and degrees of mobility that were not captured. Second, the usual activities domain was viewed as problematic as being vague or capturing adapted activities rather than those that were usual for other people. Third, the anxiety/depression domain was criticised for combining the two constructs that were seen by participants as separable. Participants stated that questions on social functioning and relationships were a key omission from the EQ-5D:

"So, you know, I wouldn't know really what box to tick because I have more than some problems in walking about, I can't walk at all and, err yeah, but I'm not confined to bed so I'm in-between there so yeah I wouldn't know really what to choose. Erm, I think, I would also say it's just got too much of a focus on walking, erm, because that isn't you know the only means of mobility and err you know these days you can get about in a wheelchair and err you know, your mobility isn't that restricted, it's, well it's more restricted by social barriers rather than erm medical ones but then, then it depends on the, you know, the quality of your wheelchair. (…) Erm, in terms of the other things, another one that really sticks out is the usual activities, erm, box. Because you adapt what your usual activities are to your level of ability and to your level of function so you, I would select that I have no problems with performing my usual activities cos you know, that's, everything is set up, if I wouldn't be able to do the activities then they wouldn't be my usual activities if that makes sense? So, erm, yeah, I don't think that would capture it very well erm because I would tick I have no problems with performing my usual activities. Whereas, obviously in all, most all aspects my function and ability is quite limited, so you wouldn't be expecting me to tick that box I think, erm." (P4, Adult).

HUI

The HUI was overall perceived as being more detailed than the EQ-5D and having more questions that were relevant to Duchenne, such as questions on the use of hands/fingers and communication/being understood. However, the measure also contained questions that were not relevant to Duchenne, such as vision and hearing. As with the EQ-5D, the HUI contained constructs combined that participants felt could be better separated (e.g.

“fretful, angry, irritable, anxious or depressed”). Omissions from the questionnaire noted by participants included relationships and independence (or choice and control):

“Err, it’s a bit better. Erm, yeah again like you said the anxiety one that’s all all them together again which it shouldn’t be. Err, yeah, so it’s not overly relevant there. Yeah speech, erm, you could have something about using a speech aid for example or that type of thing. Cause I do tend to use it because my voice is a bit quiet so I need that. Erm, where were we. Yeah, seven’s standardish isn’t it, I’ve seen that on lots of different questionnaires even mental health ones.” (P7, Adult).
